# Age-Related Loss of Lumbar Spinal Lordosis and Mobility *–* A Study of 323 Asymptomatic Volunteers

**DOI:** 10.1371/journal.pone.0116186

**Published:** 2014-12-30

**Authors:** Marcel Dreischarf, Laia Albiol, Antonius Rohlmann, Esther Pries, Maxim Bashkuev, Thomas Zander, Georg Duda, Claudia Druschel, Patrick Strube, Michael Putzier, Hendrik Schmidt

**Affiliations:** 1 Julius Wolff Institute, Charité – Universitätsmedizin Berlin, Berlin, Germany; 2 Centrum für Muskuloskeletale Chirurgie, Charité – Universitätsmedizin Berlin, Berlin, Germany; Georgia Regents University, United States of America

## Abstract

**Background:**

The understanding of the individual shape and mobility of the lumbar spine are key factors for the prevention and treatment of low back pain. The influence of age and sex on the total lumbar lordosis and the range of motion as well as on different lumbar sub-regions (lower, middle and upper lordosis) in asymptomatic subjects still merits discussion, since it is essential for patient-specific treatment and evidence-based distinction between painful degenerative pathologies and asymptomatic aging.

**Methods and Findings:**

A novel non-invasive measuring system was used to assess the total and local lumbar shape and its mobility of 323 asymptomatic volunteers (age: 20–75 yrs; BMI <26.0 kg/m^2^; males/females: 139/184). The lumbar lordosis for standing and the range of motion for maximal upper body flexion (RoF) and extension (RoE) were determined. The total lordosis was significantly reduced by approximately 20%, the RoF by 12% and the RoE by 31% in the oldest (>50 yrs) compared to the youngest age cohort (20–29 yrs). Locally, these decreases mostly occurred in the middle part of the lordosis and less towards the lumbo-sacral and thoraco-lumbar transitions. The sex only affected the RoE.

**Conclusions:**

During aging, the lower lumbar spine retains its lordosis and mobility, whereas the middle part flattens and becomes less mobile. These findings lay the ground for a better understanding of the incidence of level- and age-dependent spinal disorders, and may have important implications for the clinical long-term success of different surgical interventions.

## Introduction

The individual shape of the lumbar spine is an essential predictor for different lumbar degenerative pathologies and for the success of various surgical interventions [1]–[5]. Moreover, the mobility of the lumbar spine is discussed as an indicator for abnormal spinal mechanics [6], [7]. The influence of the factors age and sex on the total lumbar lordosis and the range of motion (RoM) still merits discussion, because it is essential for a patient-specific treatment, for example, with regard to patient age and disease localisation, and evidence-based distinction between painful degenerative pathologies and asymptomatic aging. Despite their influence, the impact of these factors on certain regions of the lumbar spine and its mobility, including upper, middle and lower lumbar spine, remains unknown.

Most studies on the effect of age showed that lumbar lordosis decreases or remains constant during the lifetime [8]–[21]. Only a few studies demonstrated an increase in lumbar lordosis with aging [22], [23]. Studies investigating sex-related differences in lordosis showed either a slightly greater lordosis in females or no significant dependency on sex [24]–[27]. However, several of these studies investigating age and sex were limited in their sample sizes, did not differentiate between males and females and subjects with or without low back pain. Moreover, in almost all of these studies the whole lumbar lordosis was usually described by a single angle (e.g. Cobb’s method), which strongly simplifies the complex lumbar curvature [28]–[30]. A more detailed analysis, including of different lumbar sub-regions, may reveal that the upper, middle or lower lumbar lordosis are affected specifically, for example, by aging.

In addition to the individual shape of the lumbar lordosis, it is generally accepted that the total RoM in flexion-extension is reduced with increasing age [6], [31]–[39]. However, similar to lumbar lordosis, it remains unknown, which lumbar sub-region is affected dominantly by aging in males and females. Knowledge about local changes in spinal function with aging may therefore help to optimise and adapt the treatment to the diseased spinal level.

Because detailed segmental diagnostic X-ray examinations are ethically questionable in healthy individuals and very laborious in large cohorts, a new technological approach was first identified and further developed by our group [40], which would allow analysis of the total and local lordosis as well as the spinal function (e.g. by means of RoM). Using this sophisticated, strain-gauge based tool that had been validated previously, the present study investigated the effect of age and sex on the lordosis and on the RoM for the whole lordosis as well as for the different lumbar sub-regions in asymptomatic volunteers across the adult lifespan. It was hypothesised that:

1. aging reduces the total lumbar lordosis and the total range of motion in flexion (RoF) and extension (RoE) of the lumbar spine,
2. locally, the change in lordosis and mobility with aging varies between different lumbar sub-regions,
3. the total lumbar lordosis is larger in females than in males.

## Materials and Methods

### Measuring system

The lumbar and thoraco-lumbar shape and the mobility of the spine in the sagittal plane were dynamically determined with the Epionics SPINE system (Epionics Medical GmbH, Potsdam, Germany). The system has the advantage over X-ray techniques that the volunteers are not exposed to radiation and thus several repeated measurements are possible. Furthermore, different sub-regions of the lumbar lordosis, including the lower, middle and upper lordosis, can be analysed. The high accuracy and reliability of the system was reported previously [40], [41].

**Figure 1:**
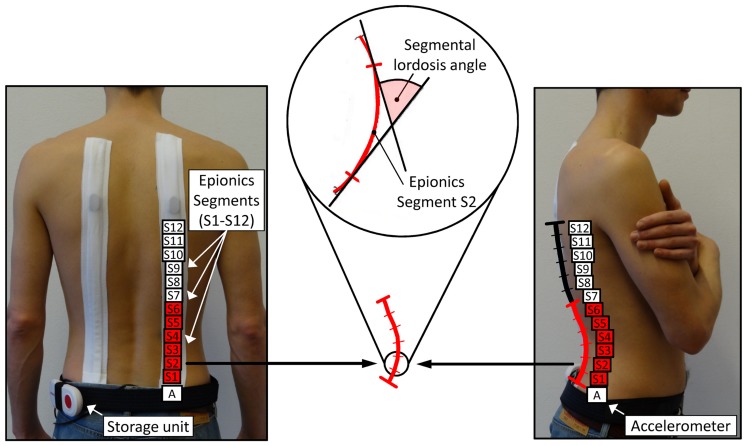
Epionics SPINE system with the positions of the Epionics segments S1–S12. On average, the lumbar lordosis is covered by the first six segments (shown in red). Middle: Schematic sketch of the definition of the determined segmental angle is shown for a single exemplary sensor unit S2.

The system consists of two flexible sensor strips, which are placed in hollow plasters onto the volunteers’ back (Fig. 1). Each of the strips are positioned paravertebrally approximately 7.5 cm from the mid-sagittal plane while the lowest part of the strips is positioned at the level of the posterior superior iliac spine (approximately first sacral vertebra). Each sensor strip consists of twelve 25-mm-long single sensor units (Epionics segments: S1–S12) containing strain-gauge technology. Each sensor unit detects the local back curvature as illustrated in Fig. 1. The data from all 12 sensors is collected at 50 Hz and saved locally on a small storage unit (12.5 cm×5.5 cm; mass: 120 g) carried on a belt, allowing free unhampered movements of the volunteer. An accelerometer is located at the lower end of each sensor strip to assess the orientation of the sensors in relation to earth’s gravitational field. A detailed description of the system has been published elsewhere [40], [41].

### Volunteers

Measurements were performed on 429 asymptomatic volunteers (198 males, 231 females). Subjects were free from acute low back pain and had no low back pain within the previous 6 months. Furthermore, volunteers had no previous spinal surgery.

Own validation and previously published studies showed that there is a significant correlation (Spearman’s correlation: 0.86, *p<0.01* [42]) between the total lumbar lordosis measured on the back and the radiologically determined lordosis in subjects with a BMI <26.0 kg/m^2^. Moreover, this value of 26.0 kg/m^2^ is considered as the normal-weight limit for subjects with an age similar to the mean age of the here investigated cohort (National Research Council, [43]) and was set to the BMI-limit in the present study.

The volunteers were classified for sex and assigned to four age groups: 20–29 years, 30–39 yrs, 40–49 yrs and>50 yrs, as was done in previous studies (e.g.: [15], [44]).

### Measurement protocol

The volunteers performed standardised motion choreographies in the sagittal plane. For guidance and standardisation, the volunteers watched a video prior to the exercise, which explained and demonstrated the exercise. Starting from a relaxed standing position, the volunteers were asked to perform a maximal upper body flexion and extension with extended knees up to six times. Before each exercise, the subjects were measured in a relaxed standing position. Each volunteer performed the movements at his or her own preferred speed.

### Data analysis

All Epionics sensor segments (S1–S12, Fig. 1) and the total lumbar lordosis were evaluated for the three investigated body positions: standing, maximal flexion and maximal extension of the upper body. The results of the left and right sensor strips were averaged. The total lumbar lordosis in standing of each volunteer was determined individually as the sum of all lordotically curved segments. The total angles for flexion and extension were calculated individually as the sum of the segments, which were identified as being lordotic during upright standing. The total RoF and RoE in the lumbar spine for each subject were calculated as the maximal or minimal angle difference with respect to standing.

### Statistical Analysis

Descriptive statistics (mean, standard error, standard deviation) were analysed using SPSS 21.0 (SPSS Inc., Chicago IL, USA). The Kolmogorov-Smirnov test was performed to evaluate the normal distribution for each investigated group. In addition, the Levene’s test was performed to test equality of variance. A two-way analysis of variance (ANOVA) with the factors age and sex was performed to evaluate the effects on the total lumbar lordosis, total RoF and total RoE. Subsequently, the mean values of the lordosis, RoF and RoE of the Epionics segments of different age groups were compared sex-specifically using a one-way ANOVA followed by post-hoc analysis using Scheffé’s test. A *p*-value <0.01 was considered as statistically significant.

### Ethics Statement

This study was approved by the Ethics Committee of the Charité – Universitätsmedizin Berlin (registry number EA4/011/10). The procedure of this study was explained to each volunteer in detail and they signed a written informed consent, which allows spinal shape determinations with the Epionics SPINE device.

## Results

### Volunteers

Due to the defined BMI-threshold of 26.0 kg/m^2^, 106 volunteers were excluded from the initial sample of 429, which resulted in a final number of 323 subjects (males: 139, females: 184). The mean values for body height, body weight and BMI for all the age cohorts are given in Table 1.

**Table 1:** Number of volunteers and mean values (standard deviation) for age, body height, body weight and body mass index.

		All	20–29 yrs	30–39 yrs	40–49 yrs	>50 yrs
**Volunteers**	**entire cohort**	323	115	70	71	67
	**female**	184	66	40	41	37
	**male**	139	49	30	30	30
**Age** **(in years)**	**entire cohort**	38.6 (14.0)	25.1 (2.7)	34.0 (3.3)	44.5 (3.2)	60.3 (7.9)
	**female**	38.4 (14.1)	24.8 (2.7)	34.0 (3.3)	44.2 (3.3)	60.8 (8.0)
	**male**	38.9 (13.7)	25.6 (2.8)	34.0 (3.3)	44.9 (3.1)	59.6 (7.8)
**Body** **height** **(in cm)**	**entire cohort**	173.0 (9.5)	173.8 (9.3)	173.2 (9.3)	172.9 (9.2)	171.5 (10.2)
	**female**	167.6 (6.9)	168.3 (7.0)	167.7 (6.4)	168.0 (7.2)	165.8 (7.0)
	**male**	180.1 (7.4)	181.2 (6.5)	180.6 (7.1)	179.6 (7.2)	178.4 (9.2)
**Body** **weight** **(in kg)**	**entire cohort**	67.6 (10.1)	66.9 (10.0)	66.7 (10.2)	68.6 (10.0)	68.6 (10.4)
	**female**	61.7 (7.5)	60.7 (6.8)	61.0 (8.2)	62.8 (7.6)	62.8 (7.7)
	**male**	75.4 (7.5)	75.1 (7.2)	74.3 (7.2)	76.6 (6.7)	75.8 (8.8)
**BMI** **(in kg/m^2^)**	**entire cohort**	22.5 (2.0)	22.0 (1.9)	22.2 (2.2)	22.9 (2.0)	23.2 (1.8)
	**female**	21.9 (2.1)	21.4 (1.9)	21.7 (2.3)	22.2 (2.1)	22.8 (1.9)
	**male**	23.2 (1.7)	22.9 (1.7)	22.8 (1.9)	23.7 (1.4)	23.7 (1.4)

### Total and local lumbar lordosis during standing

**Figure 2:**
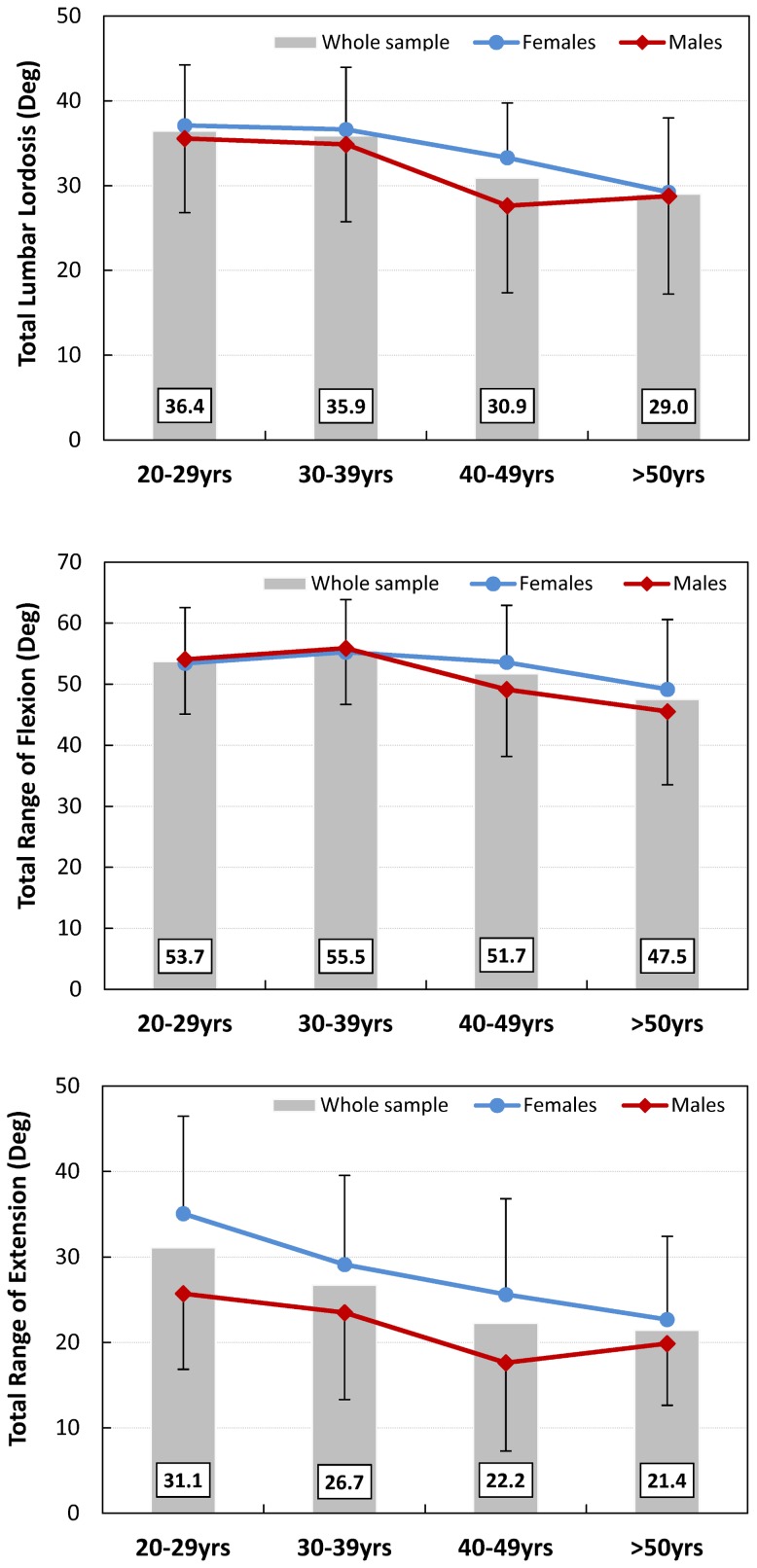
Mean values of the total lumbar lordosis (top), total range of flexion (middle) and total range of extension (bottom) in all four investigated age groups for the whole cohort (grey columns). The red lines represent males and the blue lines females. Error bars represent the standard deviation.

The Kolmogorov-Smirnov test showed that the lumbar lordosis followed a normal distribution for both sexes. Two-way ANOVA demonstrated that the total lumbar lordosis was only significantly associated with age, but not with sex (Table 2). For the whole sample, this reduction of the total lordosis with age occurred with each consecutive age group (Fig. 2 top; Table 3). There was a significant reduction of approximately 7.4° (≈20%) between the youngest and the oldest cohort. This decrease was more evident in females, who showed a significant reduction of 7.9°, than in males with 6.7°, which was only close to significant (*p = 0.034*). For both sexes, there was only a small lordosis reduction between 20–29 yrs and 30–39 yrs. In the following aging process, females showed a continuous decrease, while the reduction in males mostly occurred between the ages 30–39 yrs and 40–49 yrs. The smallest total lordosis in males was measured within the 40–49 yrs group. Post-hoc comparison demonstrated no statistical differences between the 40–49 yrs and>50 yrs age groups (*p = 0.977*) in males, whereas there was a significant reduction of the lordosis between the 20–29 yrs and the 40–49 yrs age groups (*p = 0.009*).

**Table 2:** Results of two-way analysis of variance (ANOVA) for age and sex for each of the three dependent variables: lumbar lordosis, range of flexion and range of extension.

	*p*-value	*Partial Eta-squared*
**Total lumbar lordosis**		
Age	**<0.001** *	0.123
Sex	0.017	0.018
Age×Sex	0.287	0.012
**Total range of flexion**		
Age	**<0.001** *	0.080
Sex	0.134	0.007
Age×Sex	0.212	0.014
**Total range of extension**		
Age	**<0.001** *	0.132
Sex	**<0.001** *	0.088
Age×Sex	0.188	0.015

*Statistically significant (p<0.01).

**Table 3:** Mean total lumbar lordosis (standard error; standard deviation) for investigated age groups.

	Parameter	20–29 yrs	30–39 yrs	40–49 yrs	>50 yrs	*ANOVA* *p*-value*	*Post-hoc:* *20–29* *yrs* *vs.>50* yrs**
**Entire** **cohort** **(n = 323)**	**Total Lumbar** **Lordosis**	36.4 (0.7; 7.9)	35.9 (1.0; 8.1)	30.9 (1.0; 8.7)	29.0 (1.2; 10.0)	**<0.001**	**<0.001**
	**S1 Lordosis**	5.6 (0.2; 2.4)	5.8 (0.3; 2.6)	5.4 (0.4; 3.2)	5.2 (0.4; 3.1)	0.599	0.752
	**S2 Lordosis**	6.3 (0.2; 2.3)	6.3 (0.3; 2.3)	5.5 (0.3; 2.5)	5.4 (0.4; 2.9)	0.020	0.085
	**S3 Lordosis**	7.3 (0.2; 2.1)	7.0 (0.3; 2.3)	6.0 (0.3; 2.3)	5.6 (0.3; 2.5)	**<0.001**	**<0.001**
	**S4 Lordosis**	6.7 (0.2; 1.8)	6.1 (0.2; 2.0)	5.0 (0.3; 2.3)	4.5 (0.3; 2.1)	**<0.001**	**<0.001**
	**S5 Lordosis**	5.1 (0.2; 1.8)	4.7 (0.2; 1.8)	3.7 (0.2; 2.1)	3.2 (0.2; 1.9)	**<0.001**	**<0.001**
	**S6 Lordosis**	3.2 (0.2; 1.8)	3.2 (0.2; 1.6)	2.5 (0.2; 2.0)	2.3 (0.2; 2.0)	**0.001**	0.015
**Males** **(n = 139)**	**Total** **Lumbar** **Lordosis**	35.5 (1.2; 8.7)	34.9 (1.7; 9.1)	27.6 (1.9; 10.2)	28.8 (2.1; 11.6)	**0.001**	0.034
	**S1 Lordosis**	6.6 (0.3; 2.2)	7.0 (0.6; 3.3)	7.2 (0.7; 3.8)	6.7 (0.7; 3.7)	0.881	0.999
	**S2 Lordosis**	7.2 (0.3; 2.3)	6.9 (0.5; 2.9)	6.3 (0.6; 3.1)	6.5 (0.6; 3.3)	0.576	0.801
	**S3 Lordosis**	7.6 (0.3; 2.3)	7.1 (0.5; 2.7)	5.7 (0.4; 2.5)	6.0 (0.5; 2.6)	**0.003**	0.065
	**S4 Lordosis**	6.3 (0.3; 2.0)	5.7 (0.4; 2.2)	4.0 (0.4; 2.0)	4.1 (0.4; 2.1)	**<0.001**	**<0.001**
	**S5 Lordosis**	4.4 (0.3; 1.8)	4.1 (0.3; 1.9)	2.3 (0.3; 1.8)	2.4 (0.4; 2.0)	**<0.001**	**<0.001**
	**S6 Lordosis**	2.4 (0.2; 1.6)	2.6 (0.3; 1.8)	1.1 (0.3; 1.7)	1.3 (0.4; 2.0)	**0.001**	0.075
**Females** **(n = 184)**	**Total** **Lumbar** **Lordosis**	37.1 (0.9; 7.2)	36.6 (1.2; 7.3)	33.3 (1.0; 6.4)	29.2 (1.4; 8.8)	**<0.001**	**<0.001**
	**S1 Lordosis**	4.9 (0.3; 2.3)	4.9 (0.2; 1.3)	4.2 (0.3; 2.0)	3.9 (0.3; 1.9)	0.041	0.119
	**S2 Lordosis**	5.7 (0.3; 2.1)	5.8 (0.3; 1.7)	4.9 (0.3; 1.8)	4.4 (0.4; 2.2)	**0.003**	0.016
	**S3 Lordosis**	7.1 (0.2; 1.9)	6.9 (0.3; 1.9)	6.2 (0.3; 2.2)	5.2 (0.4; 2.4)	**<0.001**	**0.001**
	**S4 Lordosis**	6.9 (0.2; 1.6)	6.4 (0.3; 1.9)	5.8 (0.3; 2.2)	4.8 (0.3; 2.1)	**<0.001**	**<0.001**
	**S5 Lordosis**	5.7 (0.2; 1.6)	5.1 (0.2; 1.5)	4.8 (0.2; 1.5)	3.9 (0.3; 1.6)	**<0.001**	**<0.001**
	**S6 Lordosis**	3.9 (0.2; 1.6)	3.8 (0.2; 1.3)	3.6 (0.2; 1.4)	3.1 (0.3; 1.6)	0.118	0.133

All measurements are in degrees.

Bold values indicate statistical significance (p<0.01).

*p-values base on one-way ANOVA. **Post-hoc comparison using Scheffé’s test.

**Figure 3:**
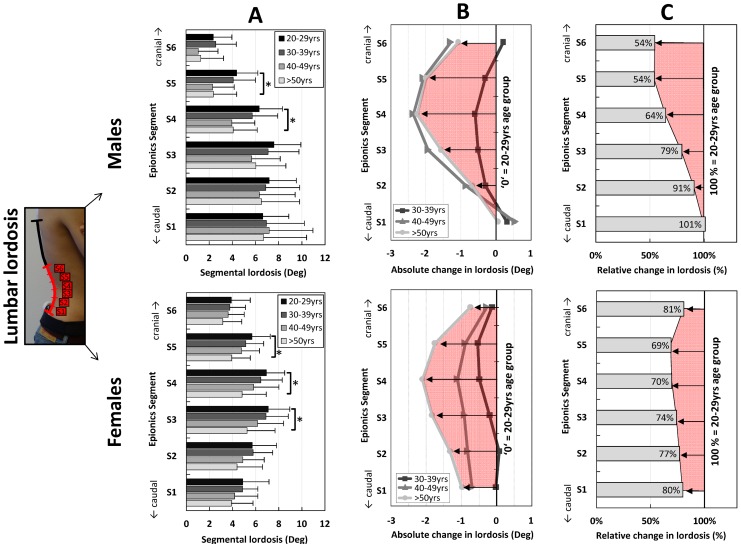
Mean values of the segmental lordosis for the Epionics segments S1 to S6 in all investigated age groups (**A**). Males (above) and females (below) are shown separately. Error bars represent the standard deviation. (**B**): Absolute change in segmental lordosis for the Epionics segments S1 to S6 in all investigated age groups in relation to the youngest cohort (20–29 yrs) for males (above) and females (below) separately. The youngest cohort is normalised to ‘zero’ as a reference. The red area highlights the pattern of the absolute change between the oldest and youngest cohorts. (**C**): Relative change in segmental lordosis for the Epionics segments S1 to S6 between the oldest and youngest age groups for males (above) and females (below) separately. The youngest cohort is normalised to 100% as a reference. Values indicate the percentage of lordosis that the oldest cohort possesses in relation to the youngest cohort. The red area highlights the pattern of the relative changes between the oldest and youngest cohorts.

**Figure 4:**
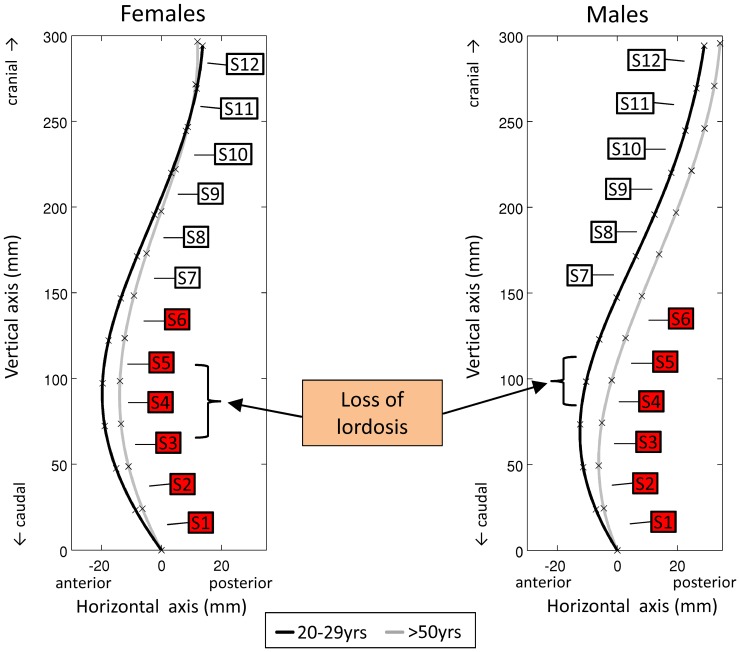
Age-related postural adaptations of the 12 Epionics segments between the oldest and youngest age cohorts for females (left) and males (right).

On average, the first six Epionics segments (S1–S6) were lordotic in the present cohort (Figs. 1, 3 A) and are therefore presented here. Epionics segments above those were part of the thoraco-lumbar transition and lower thoracic kyphosis. Locally for both sexes, the greatest loss of lordosis occurred in the middle part of the lordosis with a tendency towards the upper part. Generally, less reduction was observed towards the thoraco-lumbar and lumbo-sacral transitions (Fig. 3B; Table 3). The largest absolute loss of lordosis between the youngest and oldest cohorts occurred in the Epionics segment S4 with values of 2–3°. The lower segments S1 and S2 and the upper segment S6 showed no significant differences for the whole sample, nor for males and females separately. For the relative change, the lordosis of the youngest cohort was set to 100% in Fig. 3 C. In males, the largest relative reduction of the lordosis occured in S6 (to 54% of the reference) with continuously less reduction towards the most caudal segment S1 (to 101%). In females, the largest relative reduction occurred in S5 (to 69%), with continuously less reduction to the most caudal segment S1 (to 80%) and furthermore less in S6 (to 81%). These local changes in males and females led to a characteristic change between the ‘young’ and the ‘old’ lordosis as illustrated in Fig. 4.

### Total and local range of flexion and extension during standing

The Kolmogorov-Smirnov test showed that the RoF and RoE were normally distributed in male and female cohorts. The total RoF and RoE were significantly associated with age (Table 2). Moreover, the total RoE, but not the total RoF, was significantly associated with sex.

For the whole sample as well as for males and females separately, the total RoF showed no significant difference between the 20–29 yrs and 30–39 yrs age cohorts, but a consecutive decrease for the subsequent age groups (Fig. 2 middle; Table 4). Although significant, the total RoF was reduced by only approximately 12% (6.2°) when comparing the youngest and oldest cohorts of the whole sample. This decrease was only significant for males, with a loss of 8.6°. In females, the reduction was only 4.2°.

**Table 4:** Mean range of flexion (RoF) and range of extension (RoE) (standard error; standard deviation) for investigated age groups.

	Parameter	20–29 yrs	30–39 yrs	40–49 yrs	>50 yrs	*ANOVA* *p*-value*	*Post-hoc:* *20–29* *yrs* *vs.>50* yrs**
**Entire** **cohort** **(n = 323)**	**Total range** **of flexion**	53.7 (0.8; 9.0)	55.5 (1.1; 8.8)	51.7 (1.2; 10.2)	47.5 (1.4; 11.8)	**<0.001** *	**0.001** *
	**RoF S1**	6.6 (0.2; 2.5)	7.3 (0.3; 2.7)	7.3 (0.4; 3.3)	7.1 (0.3; 2.5)	0.270	0.720
	**RoF S2**	8.8 (0.2; 2.2)	9.0 (0.3; 2.3)	8.2 (0.3; 2.3)	7.8 (0.3; 2.3)	**0.006** *	0.046
	**RoF S3**	10.9 (0.2; 2.3)	10.6 (0.3; 2.4)	9.6 (0.3; 2.2)	8.7 (0.3; 2.4)	**<0.001** *	**<0.001** *
	**RoF S4**	10.2 (0.2; 2.3)	9.7 (0.3; 2.1)	9.0 (0.2; 2.1)	7.9 (0.3; 2.3)	**<0.001** *	**<0.001** *
	**RoF S5**	7.8 (0.2; 2.2)	7.7 (0.2; 1.9)	7.5 (0.2; 2.0)	6.4 (0.3; 2.1)	**<0.001** *	**<0.001** *
	**RoF S6**	4.9 (0.2; 1.9)	5.3 (0.2; 1.7)	5.4 (0.3; 2.1)	4.8 (0.3; 2.1)	0.211	0.986
	**Total range** **of extension**	31.1 (1.1; 11.3)	26.7 (1.3; 10.6)	22.2 (1.4; 11.5)	21.4 (1.1; 8.8)	**<0.001** *	**<0.001** *
	**RoE S1**	5.4 (0.3; 3.3)	4.7 (0.3; 2.4)	4.2 (0.4; 3.1)	3.9 (0.3; 2.8)	**0.007** *	0.017
	**RoE S2**	7.0 (0.3; 2.9)	5.9 (0.3; 2.6)	4.5 (0.3; 2.8)	3.8 (0.3; 2.3)	**<0.001** *	**<0.001** *
	**RoE S3**	7.9 (0.3; 3.0)	6.5 (0.3; 2.8)	4.6 (0.4; 3.1)	3.9 (0.3; 2.2)	**<0.001** *	**<0.001** *
	**RoE S4**	6.2 (0.3; 3.0)	5.0 (0.3; 2.5)	3.6 (0.3; 2.9)	3.4 (0.2; 2.0)	**<0.001** *	**<0.001** *
	**RoE S5**	3.4 (0.2; 2.6)	2.8 (0.2; 2.0)	2.5 (0.3; 2.2)	2.7 (0.2; 1.8)	0.038	0.311
	**RoE S6**	1.2 (0.2; 2.3)	1.3 (0.2; 2.0)	1.6 (0.2; 2.0)	2.1 (0.2; 1.8)	0.032	0.048
**Males** **(n = 139)**	**Total range** **of flexion**	54.1 (1.3; 9.0)	55.9 (1.7; 9.2)	49.1 (2.0; 10.9)	45.5 (2.2; 12.0)	**<0.001** *	**0.005**
	**RoF S1**	7.2 (0.4; 2.6)	8.1 (0.7; 3.6)	8.8 (0.7; 3.9)	7.7 (0.6; 3.1)	0.191	0.931
	**RoF S2**	9.5 (0.3; 2.3)	9.6 (0.5; 2.8)	9.2 (0.5; 2.8)	8.3 (0.5; 2.5)	0.182	0.274
	**RoF S3**	11.5 (0.3; 2.4)	11.1 (0.5; 2.6)	9.8 (0.4; 2.4)	9.2 (0.4; 2.4)	**<0.001** *	**0.002**
	**RoF S4**	10.6 (0.3; 2.3)	10.1 (0.4; 2.2)	8.6 (0.4; 2.2)	7.9 (0.4; 2.3)	**<0.001** *	**<0.001** *
	**RoF S5**	8.2 (0.3; 2.1)	8.1 (0.4; 2.1)	6.8 (0.4; 2.0)	5.9 (0.4; 2.1)	**<0.001** *	**<0.001** *
	**RoF S6**	4.9 (0.3; 1.8)	5.4 (0.4; 2.0)	4.7 (0.4; 2.2)	4.0 (0.4; 2.0)	0.072	0.334
	**Total range** **of extension**	25.7 (1.3; 8.8)	23.5 (1.9; 10.2)	17.6 (1.9; 10.3)	19.9 (1.3; 7.2)	**0.001** *	0.061
	**RoE S1**	5.3 (0.4; 2.9)	4.4 (0.4; 2.3)	4.9 (0.6; 3.5)	5.0 (0.5; 2.9)	0.601	0.973
	**RoE S2**	6.5 (0.4; 2.6)	5.3 (0.5; 2.6)	4.2 (0.5; 2.7)	4.3 (0.4; 2.1)	**<0.001** *	**0.005**
	**RoE S3**	6.8 (0.4; 2.6)	5.7 (0.5; 2.9)	3.5 (0.5; 2.5)	3.9 (0.4; 2.0)	**<0.001** *	**<0.001** *
	**RoE S4**	4.7 (0.3; 2.4)	4.2 (0.5; 2.5)	2.1 (0.4; 2.3)	2.9 (0.4; 2.0)	**<0.001** *	0.013
	**RoE S5**	2.2 (0.3; 2.1)	2.4 (0.3; 1.9)	1.3 (0.4; 2.0)	2.0 (0.4; 1.9)	0.184	0.990
	**RoE S6**	0.5 (0.3; 1.8)	1.1 (0.3; 1.6)	1.0 (0.3; 1.9)	1.4 (0.3; 1.7)	0.160	0.204
**Females** **(n = 184)**	**Total range** **of flexion**	53.4 (1.1; 9.1)	55.2 (1.4; 8.6)	53.6 (1.5; 9.3)	49.2 (1.9; 11.5)	0.041	0.199
	**RoF S1**	6.2 (0.3; 2.3)	6.6 (0.2; 1.5)	6.3 (0.3; 2.2)	6.6 (0.3; 1.9)	0.633	0.788
	**RoF S2**	8.3 (0.2; 1.9)	8.5 (0.3; 1.7)	7.5 (0.3; 1.7)	7.4 (0.3; 2.1)	0.012	0.151
	**RoF S3**	10.4 (0.3; 2.1)	10.2 (0.3; 2.1)	9.4 (0.3; 2.1)	8.4 (0.4; 2.4)	**<0.001** *	**<0.001** *
	**RoF S4**	9.9 (0.3; 2.2)	9.5 (0.3; 2.2)	9.3 (0.3; 2.0)	7.9 (0.4; 2.4)	**<0.001** *	**<0.001** *
	**RoF S5**	7.6 (0.3; 2.3)	7.5 (0.3; 1.8)	8.0 (0.3; 1.9)	6.9 (0.3; 2.0)	0.126	0.392
	**RoF S6**	4.9 (0.3; 2.0)	5.2 (0.2; 1.5)	5.8 (0.3; 2.0)	5.4 (0.3; 2.0)	0.121	0.677
	**Total range of extension**	35.1 (1.4; 11.4)	29.1 (1.6; 10.4)	25.6 (1.8; 11.2)	22.7 (1.6; 9.7)	**<0.001** *	**<0.001** *
	**RoE S1**	5.3 (0.4; 3.6)	4.9 (0.4; 2.5)	3.8 (0.4; 2.8)	3.0 (0.4; 2.3)	**<0.001** *	**0.002**
	**RoE S2**	7.4 (0.4; 3.1)	6.3 (0.4; 2.6)	4.7 (0.4; 2.8)	3.4 (0.4; 2.3)	**<0.001** *	**<0.001** *
	**RoE S3**	8.7 (0.4; 3.0)	7.1 (0.4; 2.7)	5.5 (0.5; 3.2)	4.0 (0.4; 2.5)	**<0.001** *	**<0.001** *
	**RoE S4**	7.3 (0.4; 2.9)	5.5 (0.4; 2.4)	4.8 (0.4; 2.8)	3.7 (0.3; 1.9)	**<0.001** *	**<0.001** *
	**RoE S5**	4.3 (0.3; 2.6)	3.2 (0.3; 2.1)	3.3 (0.3; 2.0)	3.3 (0.2; 1.5)	0.026	0.192
	**RoE S6**	1.7 (0.3; 2.5)	1.4 (0.4; 2.2)	2.1 (0.3; 2.1)	2.7 (0.3; 1.8)	0.067	0.205

All measurements are in degrees.

Bold values indicate statistical significance (p<0.01).

*p-values base on one-way ANOVA. **Post-hoc comparison using Scheffé’s test.

**Figure 5:**
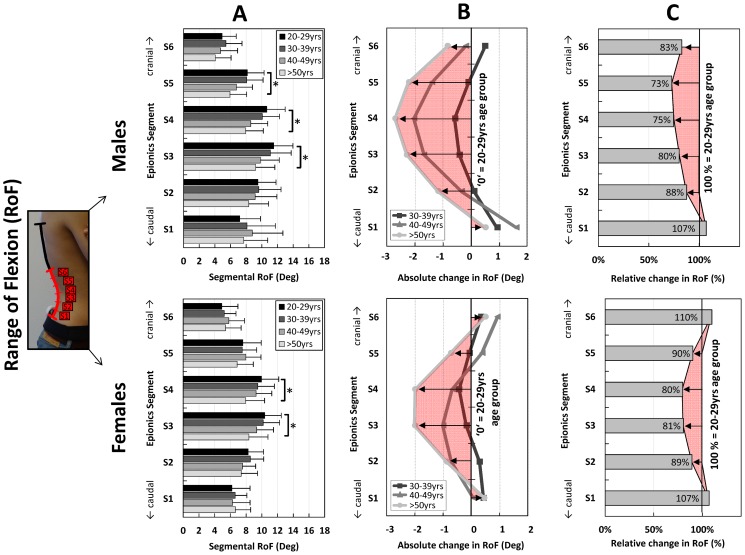
Mean values of the segmental range of flexion (RoF) for the Epionics segments S1 to S6 in all investigated age groups (**A**). Males (above) and females (below) are shown separately. Error bars represent the standard deviation. (**B**): Absolute change in the segmental RoF for the Epionics segments S1 to S6 in all investigated age groups in relation to the youngest cohort (20–29 yrs) for males (above) and females (below) separately. The youngest cohort is normalised to a value of ‘zero’ as a reference. The red area highlights the pattern of the absolute change between the oldest and youngest cohort. (**C**): Relative change in the segmental RoF for the Epionics segments S1 to S6 between oldest and youngest age groups for males (above) and females (below) separately. The youngest cohort is normalised to 100% as a reference. Values indicate the percentage of the RoF the oldest cohort possesses in relation to the youngest cohort. The red area highlights the pattern of the relative changes between the oldest and youngest cohorts.

Males and females showed similar local patterns for the reduction of the RoF with increasing age. This local reduction was most dominantly in the middle part (S3, S4) of the lumbar region (Fig. 5 A, B; Table 4). In general, the Epionics segments close to the thoraco-lumbar and lumbo-sacral transition (S1, S6) showed non-significant absolute changes with increasing age. Males tended to display a slightly greater reduction relative to the youngest cohort than females, with the greatest loss in segment S5 (to 73% of the reference; Fig. 5 C). In females, the largest relative reduction occurred in S4 (to 80%).

The total RoE of the whole sample was reduced for each consecutive age cohort and was significantly decreased by approximately 31% when comparing the youngest and oldest cohort (Fig. 2 bottom; Table 4). The loss of the RoE was more pronounced and only significant in females (12.4°). Males showed a smaller reduction of only 5.8°. Independent of age, males had a smaller total RoE than females.

**Figure 6:**
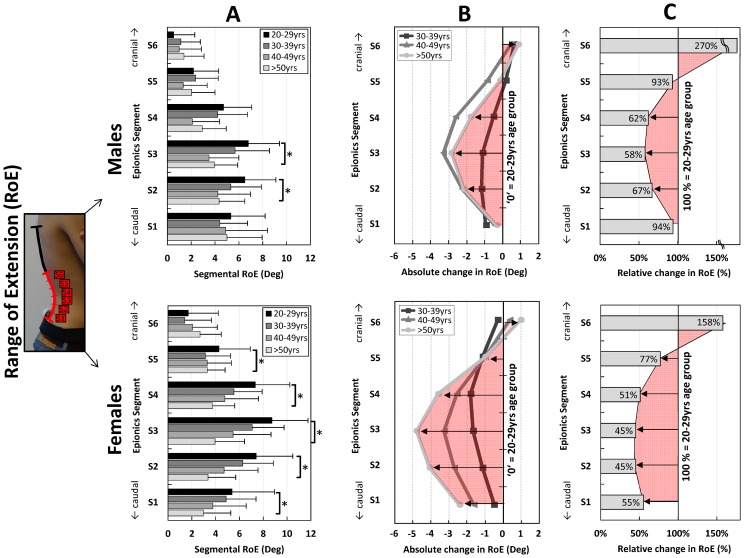
Mean values of the segmental range of extension (RoE) for the Epionics segments S1 to S6 in all investigated age groups (**A**). Males (above) and females (below) are shown separately. Error bars represent the standard deviation. (**B**): Absolute change in the segmental RoE for the Epionics segments S1 to S6 in all investigated age groups in relation to the youngest cohort (20–29 yrs) for males (above) and females (below) separately. The youngest cohort is normalised to ‘zero’ as a reference. The red area highlights the pattern of the absolute change between the oldest and youngest cohort. (**C**): Relative change in the segmental RoE for the Epionics segments S1 to S6 between the oldest and youngest age groups for males (above) and females (below) separately. The youngest cohort is normalised to 100% as a reference. Values indicate the percentage of the RoE the oldest cohort possesses in relation to the youngest cohort. The red area highlights the pattern of the relative changes between oldest and youngest cohorts.

For the local RoE, males and females both showed the largest absolute reduction in the middle part of the lordosis (S3; Fig. 6 A, B) and, similar to the local RoF, less reduction towards the transition zones. However, in females, the absolute and relative reductions were more pronounced in the middle and lower Epionics segments (Fig. 6 C). Males showed no significant and only small relative changes in the Epionics segment S1. In both sexes only small non-significant changes in the RoE occurred in segments close to the thoraco-lumbar transition (S5, S6).

## Discussion

This study investigated the effect of age and sex on the lordosis and the RoM of the whole lumbar spine as well as for different lumbar sub-regions in asymptomatic volunteers across the adult lifespan. The results of the present study emphasise the importance of the factor age on the lumbar lordosis and the RoM. We demonstrated that the age-related changes in the lordosis and the RoM differ between men and women and are strongly level dependent. The lordosis and RoM in the middle part of the lumbar spine are dominantly reduced with aging, with less reduction towards the lumbo-sacral and thoraco-lumbar transitions. The sex affects only the RoE.

The loss of total lordosis with aging as demonstrated in this study (Fig. 4) is in agreement with measurements in the literature [9], [11], [17] and corroborates our first hypothesis. This study provides evidence that this aging process is not uniform throughout lifespan and differs between males and females. In both sexes, the decrease of lordosis appears only marginal between 20–29 yrs and 30–39 yrs. While in females the process of aging is subsequently more continuous, the loss of lordosis in males mostly occurs between the 30–39 yrs and 40–49 yrs age groups. This discontinuous loss of lordosis explains why in some studies, in which only cohorts older than 40 years with no young control group were investigated, no significant loss of lordosis was found [14], [20]. In the present study, a high inter-subject variability was found, which necessitates a sufficient cohort size with a homogeneous composition to detect these age effects. Furthermore, in the present study, asymptomatic subjects were investigated, whereas in other studies subjects with acute or chronic low back pain participated. However, the change in lordosis during aging differs between asymptomatic and symptomatic subjects, because the latter may already have, for example, a flat sagittal alignment or spinal diseases that affected the spinal curvature during an earlier stage of life [1]–[3]. Similar to the lordosis in standing, aging is also the crucial factor for a reduction in total RoM, especially in extension, where it is reduced by 31% between the oldest and youngest cohorts. This is consistent with previous studies [38], [39], [45].

In opposite to our third hypothesis, the lumbar lordosis was not significantly different between both sexes, which is in agreement with several studies [9], [14], [24], [26], however in opposite to other investigations [12], [46]. The present study suggests that the difference in lordosis between men and women is small and varies between age groups. This might partly explain why studies with varying cohort sizes and different mean ages show contradictory results. Furthermore, in this sample, only subjects with a BMI <26.0 kg/m^2^ participated, which resulted in a mean BMI of 22.5 kg/m^2^. Therefore, the impact of being overweight or obese was not investigated.

Currently, a detailed investigation of the age effect on certain regions of the lordosis and its motion is lacking in the literature. In accordance with our second hypothesis, the lower Epionics segments were less affected by aging than the middle segments, which characteristically changes the total lordosis and ‘concentrates’ the lordotic shape of the lumbar spine to the lower segments. Only one radiological study on a small cohort supports our findings of a significant correlation between age and lordosis loss restricted to the middle lordosis (L3–4) [9]. Only a trend was observed in the adjacent segments L2–3 and L4–5, and no significant influence was found in L5–S1. Previous studies reported a close relationship between the morphology of the pelvis, as, for example, characterised by the pelvic incidence [47], and the degree of total lumbar lordosis [25], [26], [48], [49]. The level specific changes in lumbar lordosis during aging suggest that different parts of the lumbar spine may substantially change their relationship to the individual pelvic incidence.

In analogy to the aging process of the lordosis, the RoM characteristically changes with age. The RoM in the middle lumbar lordosis also decreases, whereas the RoM next to the thoracic and sacral transitions only shows a small change. Therefore, not only the lower lumbar lordosis but also its mobility is preserved during aging. These facts may have important implications for the spinal loading and the prevalent degeneration process in the lower lumbar spine during life, and could help to understand the mechanical challenges the lower lumbar spine has to withstand. However, these results also have consequences for the treatment of degenerative spinal diseases. Because the shape and motion differently change for certain regions of the lumbar spine, an age- and lumbar level-specific treatment may be important for long-term patient satisfaction.

This study emphasises that a reduction of the lordosis in symptomatic subjects with a severe, painful degenerated lumbar spine partly consists of a natural adaptive process during aging, which also occurs in asymptomatic subjects. Knowledge of this physiological loss of lordosis in asymptomatic individuals may, however, be essential for surgical reconstruction concepts of the sagittal alignment of the spine. In these concepts, the degree of lordosis is estimated mostly with the help of the individual pelvic incidence of the patient, which is assumed to be independent of posture and age (e.g.: lumbar lordosis  =  pelvic incidence ±9°; [50]). Because of the physiological loss of lordosis with aging, the relationship between the lumbar lordosis and pelvic incidence appears to also be dependent on age. Therefore, an optimal patient-specific reconstruction may require an age dependent estimation of the lordosis.

Although the results presented here are consistent with radiological measurements, it should be noted that the Epionics SPINE system determines the curvature of the back and not directly the shape of the spine. Multiple studies previously demonstrated that the curvature and motion measured on the back and the spine significantly correlate with each other [42], [51], [52]. In our own preliminary validation studies, we could additionally show that the correlation between the back and spinal shape is poor in overweight and obese persons, which limits this study to normal-weight subjects (BMI <26.0 kg/m^2^). However, this study investigated the spinal shape and motion of a large asymptomatic cohort for which a radiological study design is ethically not supportable. Furthermore, this study is limited by investigating the motion only in the sagittal plane, although the motion of the lumbar spine in other anatomical planes such as during axial rotation and lateral bending might be affected by aging as well.

In conclusion, this study characterises the adaptive response of the lumbar spinal shape and its mobility as a function of age in asymptomatic males and females. While the lower part of the lumbar spine retains its lordosis and mobility, the middle part flattens and becomes less mobile. This may have important implications for the clinical long-term success of different surgical interventions, for instance for the surgical reconstruction of the sagittal alignment. Furthermore, the results can help to better understand the incidence of level- and age-dependent spinal disorders, and are essential for patient specific treatments and an evidence-based distinction between painful degenerative pathologies and asymptomatic aging.
